# DNA methylation patterns are influenced by *Pax3* :: *Foxo1* expression and developmental lineage in rhabdomyosarcoma tumours forming in genetically engineered mouse models

**DOI:** 10.1002/path.6386

**Published:** 2025-01-15

**Authors:** Wenyue Sun, Stephen M Hewitt, Hollis Wright, Charles Keller, Frederic G Barr

**Affiliations:** ^1^ Laboratory of Pathology, Center for Cancer Research NCI Bethesda MD USA; ^2^ Children's Cancer Therapy Development Institute Hillsboro OR USA

**Keywords:** rhabdomyosarcoma, fusion gene, DNA methylation, mutation, PAX3::FOXO1, muscle development, mouse model

## Abstract

Rhabdomyosarcoma (RMS) is a family of phenotypically myogenic paediatric cancers consisting of two major subtypes: fusion‐positive (FP) RMS, most commonly involving the *PAX3*::*FOXO1* fusion gene, formed by the fusion of paired box 3 (*PAX3*) and forkhead box O1 (*FOXO1*) genes, and fusion‐negative (FN) RMS, lacking these gene fusions. In humans, DNA methylation patterns distinguish these two subtypes as well as mutation‐associated subsets within these subtypes. To investigate the biological factors responsible for these methylation differences, we profiled DNA methylation in RMS tumours derived from genetically engineered mouse models (GEMMs) in which various driver mutations were introduced into different myogenic lineages. Our unsupervised analyses of DNA methylation patterns in these GEMM tumours yielded two major clusters, corresponding to high and no/low expression of *Pax3*::*Foxo1*, which mirrored the results for human FP and FN RMS tumours. Two distinct methylation‐defined subsets were found for GEMM RMS tumours with no/low *Pax3*::*Foxo1* expression: one subset enriched in *Pax7* lineage tumours and a second subset enriched in myogenic factor 5 (*Myf5*) lineage tumours. Integrative analysis of DNA methylation and transcriptomic data in mouse and human RMS revealed a common group of differentially methylated and differentially expressed genes, highlighting a conserved set of genes functioning in both human RMS models and GEMMs of RMS. In conclusion, these studies provide insight into the roles of oncogenic fusion proteins and developmental lineages in establishing DNA methylation patterns in FP and FN RMS respectively. © 2025 The Author(s). *The Journal of Pathology* published by John Wiley & Sons Ltd on behalf of The Pathological Society of Great Britain and Ireland. This article has been contributed to by U.S. Government employees and their work is in the public domain in the USA.

## Introduction

Rhabdomyosarcoma (RMS) is the most common soft tissue tumour in children and adolescents [1]. Genetically, RMS is classified into fusion‐positive (FP) and fusion‐negative (FN) subtypes, based on the presence or absence of characteristic fusion genes. FP RMS is characterised by the presence of the paired box 3 and forkhead box O1 (*PAX3*::*FOXO1*) gene fusion or paired box 7 and forkhead box O1 (*PAX7*::*FOXO1*) gene fusion and is usually associated with alveolar RMS histology, whereas FN RMS lacks these gene fusions and is usually associated with embryonal RMS histology [2, 3, 4, 5]. Genome‐wide DNA methylation studies provided initial evidence for an association of DNA methylation patterns with histological subtypes [6, 7, 8]. We previously demonstrated distinct DNA methylation profiles in FP and FN RMS and in mutation‐associated subsets of the FP and FN RMS categories [9, 10].

RMS resembles developing skeletal muscle and is postulated to originate in muscle progenitor cells, although more recent evidence suggests that some RMS subsets may originate from aberrant development of non‐myogenic cells [11, 12, 13]. Previously we reported the creation and characterisation of genetically engineered mouse models (GEMMs) of RMS with activation of *Pax3*::*Foxo1* gene fusion expression and/or inactivation of patched 1 (*Ptch1*), tumour protein 53 (*Trp53*), and/or RB transcriptional corepressor 1 (*Rb1*) in several prenatal or postnatal myogenic lineages [14, 15, 16, 17]. Our convolutional neural network‐based differential diagnosis model determined that tumours from the GEMMs of RMS were within the spectrum of human RMS tumours [18].

Numerous studies have highlighted that DNA methylation is conserved across mice and humans and, furthermore, that DNA methylation in promoter regions and gene bodies is implicated in the regulation of gene expression in both mice and humans [19, 20, 21, 22]. In this study, we examined the DNA methylation patterns of *n* = 31 tumours from GEMMs of RMS and investigated their relationship with developmental lineages and mutational changes. We also compared the DNA methylation and expression patterns in GEMM tumours to the patterns in human RMS tumours. These results shine a light on the contribution of both cell of origin and mutational changes to the establishment of genome‐wide DNA methylation patterns in RMS.

## Materials and methods

### Animal models

GEMMs of RMS were generated as described previously [14, 15, 16, 17]. These models were generated to introduce driver events into specific muscle cell types in foetal and postnatal development, including the embryonic muscle lineage (*Pax3*), embryonic and foetal muscle lineages [myogenic factor 5 (*Myf5*); myogenic factor 6 (*Myf6*)], and postnatal satellite cell lineage (*Pax7*). These models incorporated mutational events including the *Pax3*::*Foxo1* fusion and inactivation of *Ptch1*, *Trp53*, and/or *Rb1* (Table 1).

**Table 1:** Characteristics of genetically engineered mouse models (GEMMs) of rhabdomyosarcoma (RMS) used in DNA methylation study.

Sample	Model	DNA Genotype
28,402	Pax3	MCre(Cre/WT) Ptch1(F1‐2m) Trp53(F2‐10/F2‐10)
22,520	Pax3	MCre(Cre/WT) Ptch1(F1‐2m) Trp53(F2‐10/F2‐10)
20,745	Pax3	MCre(Cre/WT) Ptch1(F1‐2m) Trp53(F2‐10/F2‐10)
24,107	Pax3‐P3F	MCre(Cre/WT) Pax3(P3Fm/P3Fm) Trp53(F2‐10/F2‐10)
23,969	Pax3‐P3F	MCre(Cre/WT) Pax3(P3Fm/P3Fm) Trp53(F2‐10/F2‐10)
24,988	Pax3‐P3F	MCre(Cre/WT) Pax3(P3Fm/P3Fm) Trp53(F2‐10/F2‐10)
24,085	Myf5	Myf5(ICNm/WT) Ptch1(F1‐2m/WT) Trp53(F2‐10/DEL2‐10)
24,055	Myf5	Myf5(ICNm/WT) Ptch1(F1‐2m/WT) Trp53(F2‐10/F2‐10)
20,938	Myf5	Myf5(ICNm/WT) Ptch1(F1‐2m/WT) Trp53(F2‐10/F2‐10)
23,478	Myf5	Myf5(ICNm/WT) Ptch1(F1‐2m/WT) Trp53(F2‐10/F2‐10) ROSA26(LUSAP)
24,014	Myf5‐P3F	Myf5(ICNm/WT) Pax3(P3Fm/P3Fm) Trp53(F2‐10/F2‐10)
32,577	Myf6	Myf6(ICNm/WT) Trp53(F2‐10/F2‐10)
37,125	Myf6	Myf6(ICNm/WT) Trp53(F2‐10/F2‐10) Rosa26(LUSAP/Any) Ptch1(WT/WT)
21,353	Myf6	Myf6(ICNm/WT) Trp53(F2‐10/F2‐10) Rosa26(LUSeAP/WT)
35,029	Myf6	Myf6(ICNm/WT) Trp53(F2‐10/F2‐10) Rb1(Flox/Flox)
32,945	Myf6‐P3F	Myf6(ICNm/WT) Pax3(P3Fm/P3Fm) Trp53(F2‐10/F2‐10)
26,404	Myf6‐P3F	Myf6(ICNm/WT) Pax3(P3Fm/P3Fm) Trp53(F2‐10/F2‐10)
44,927	Myf6‐P3F	Myf6(ICNm/WT) Pax3(P3Fm/P3Fm) Trp53(F2‐10/F2‐10) Hairless(Skh1/WT)
38,917	Myf6‐P3F	Myf6(ICNm/WT) Pax3(P3Fm/P3Fm) Trp53(F2‐10/F2‐10) Rb1(Flox/Flox)
38,172	Myf6‐P3F	Myf6(ICNm/WT) Pax3(P3Fm/P3Fm) Trp53(F2‐10/F2‐10) Rb1(Flox/Flox) Rosa26(LUSAP/Any)
66,788	Myf6‐P3F	Myf6(ICNm/WT) Pax3(P3Fm/P3Fm) Trp53(F2‐10/F2‐10) Rosa26(LUSAPp/Any) Hairless(Skh1/Skh1)
45,065	Myf6‐P3F	Myf6(ICNm/WT) Pax3(P3Fm/P3Fm) Trp53(F2‐10/F2‐10) Rb1(Flox/Flox) Rosa26(LUSAP/WT)
33,940	Pax7	ROSA26(Yello/LUSAP) Trp53(F2‐10/F2‐10) Pax7(CreERp/WT)
34,605	Pax7	ROSA26(WT/WT) Ptch1(F1‐2m/WT) Trp53(F2‐10/F2‐10) Pax7(CreERp/WT)
31,431	Pax7‐P3F	Pax7(CreERp/WT) Pax3(P3Fm/P3Fm) Trp53(F2‐10/F2‐10) Rosa26(LUSAP/Any)
28,285	Pax7‐P3F	Pax7(CreERp/WT) Pax3(P3Fm/P3Fm) Trp53(F2‐10/F2‐10) Rosa26(LUSAPm/WT)
29,418	Pax7‐P3F	Pax7(CreERp/WT) Pax3(P3Fm/P3Fm) Trp53(F2‐10/F2‐10) Rosa26(LUSAPm/WT) ZRED‐Tg(Bgeo/WT)
35,512	Pax7‐P3F	Pax7(CreERp/WT) Pax3(P3Fm/P3Fm) Trp53(F2‐10/F2‐10) Rb1(Flox/Flox)
31,430	Pax7‐P3F	Pax7(CreERp/WT) Pax3(P3Fm/P3Fm) Trp53(F2‐10/F2‐10) Rosa26(Lusap/WT)
26,568	Pax7‐P3F	Pax3(P3Fm/P3Fm) ROSA26(WT/WT) Trp53(F2‐10/F2‐10) Pax7(CreERp/WT)
29,138	Pax7‐P3F	Pax3(P3Fm/P3Fm) Trp53(F2‐10/F2‐10) Pax7(CreERp/WT)

### Mouse tumour processing

Snap‐frozen mouse tumours (*n* = 31) were fixed in buffered ethanol fixative BE70, and paraffin‐embedded tissue blocks were prepared [23]. Haematoxylin and eosin‐stained slides were reviewed, and regions of >85% viable tumour were marked and selected for coring [24]. DNA was extracted from these cores using the DNeasy Blood and Tissue Kit (Qiagen, Hilden, Germany) [25].

### Genome‐wide DNA methylation analysis

Genomic DNA from GEMM tumours was analysed on the Infinium Mouse Methylation BeadChip (Illumina, San Diego, CA, USA) (GEO: GSE260806). DNA methylation data from human RMS tumours generated previously on the Infinium HumanMethylation450 (HM450) BeadChip (Illumina) were included in this analysis [National Institutes of Health (NIH) database of genotypes and phenotypes (dbGaP): phs001970]. DNA methylation array data were normalised using the Subset‐Quantile Within Array Normalization (SWAN) algorithm in the minfi package (https://bioconductor.org/packages/release/bioc/html/minfi.html, last accessed 22 April 2024) [26]. Hierarchical cluster analysis and *t*‐distributed Stochastic Neighbor Embedding (t‐SNE) were generated using the gplots and t‐SNE packages (https://CRAN.R-project.org/package=gplots and https://CRAN.R-project.org/package=Rtsne, last accessed 22 April 2024), respectively [9, 10, 27]. Additional details are provided in Supplementary materials and methods.

### Gene expression analysis

For mouse tumours, gene expression analysis was previously performed using MouseRef‐8 Expression BeadChip (Illumina) [16, 17]. For human tumours, RNA‐seq data were obtained from the OncoGenomics database (https://pob.abcc.ncifcrf.gov/cgibin/JK, last accessed 22 April 2024) [28]. Additional details are provided in Supplementary materials and methods.

### Genomic annotations

IlluminaMouseMethylationanno.12.v1.mm10, IlluminaHumanMethylation450kanno.ilmn12.hg19, TxDb.Mmusculus.UCSC.mm10.knownGene, and TxDb.Hsapiens.UCSC.hg19.knownGene annotation packages (https://github.com/chiaraherzog/IlluminaMouseMethylationanno.12.v1.mm10, https://bioconductor.org/packages/IlluminaHumanMethylation450kanno.ilmn12.hg19, https://bioconductor.org/packages/TxDb.Mmusculus.UCSC.mm10.knownGene, and https://bioconductor.org/packages/TxDb.Hsapiens.UCSC.hg19.knownGene, last accessed 22 April 2024) were used. A probe was classified as within a ‘promoter’ if it was within the 1,500‐bp region upstream of the annotated transcription start site and/or the first exon of any transcript [29]. A probe was classified as in the ‘body’ region if the probe was inside any intron or any exon other than the first exon.

## Results

### DNA methylation related to mutations and myogenic lineage

**Figure 1:**
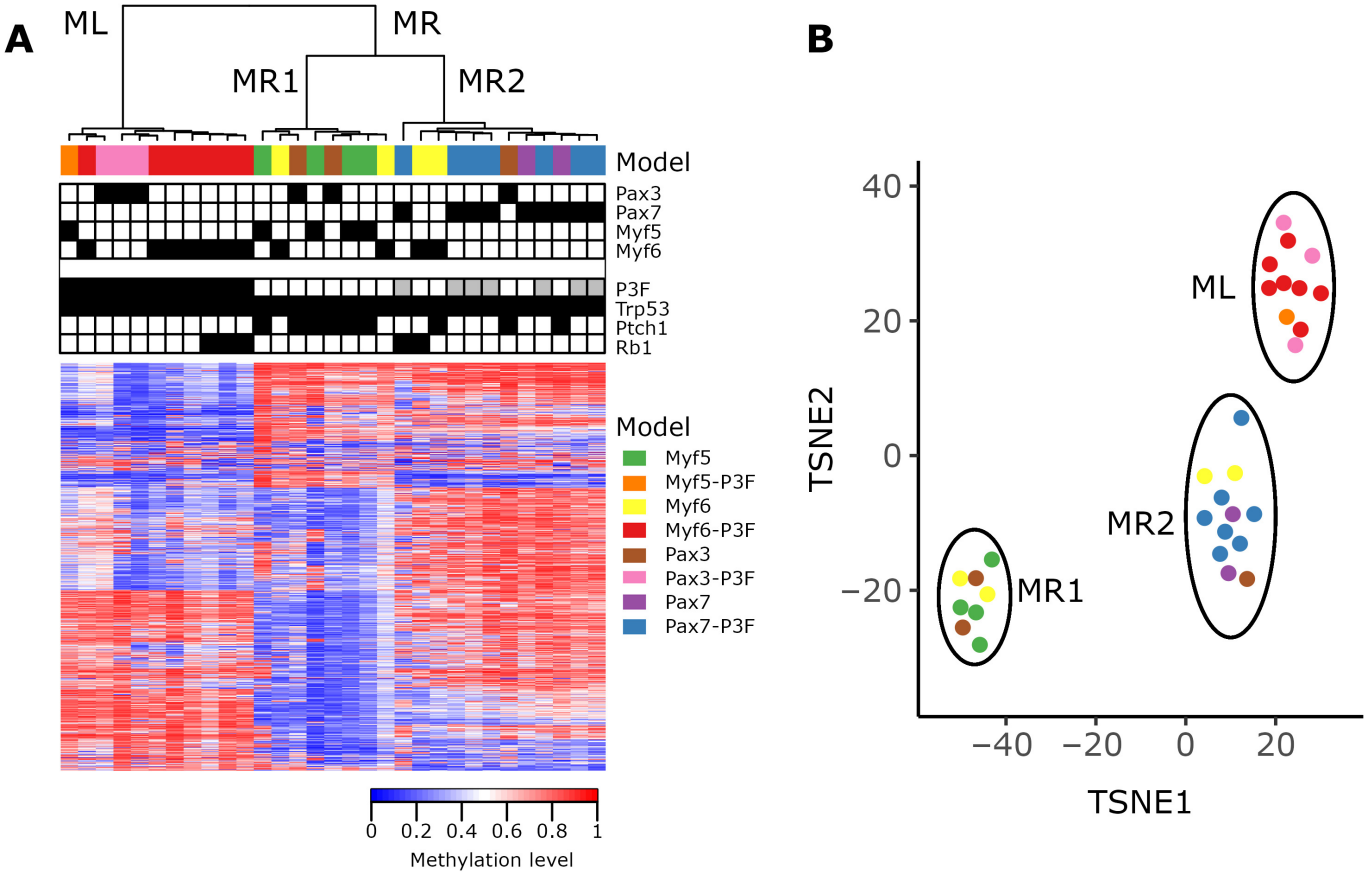
Association of lineage and mutational changes with DNA methylation in GEMMs of rhabdomyosarcoma. (A) Unsupervised hierarchical clustering of methylation profiles from *n* = 31 mouse tumours. In the heat map, rows represent probes and columns represent tumour samples. Dendrogram at top shows main clusters (ML and MR clusters) and MR subsets (MR1 and MR2). Beneath the dendrogram, the top‐row colours are based on the GEMMs defined in the ‘Model’ key. The next set of rows indicates the lineages and genetic mutations in the *n* = 31 mouse tumours. (B) t‐SNE visualisation of mouse tumours. Each sample's colour is based on the GEMMs defined in panel (A), and groupings are circled and labelled as determined in panel (B).

We determined DNA methylation profiles in *n* = 31 mouse tumours derived from GEMMs of RMS (Table 1) on an array‐based mouse methylation platform. Using the top 1% most variable probes, unsupervised hierarchical clustering analysis identified two main clusters that separated the mouse tumours [methylation left (ML) and methylation right (MR) clusters] and two prominent subsets within the MR cluster (MR1 and MR2) (Figure 1A). Initial examination of these clusters/subsets revealed that all GEMM tumours in ML clusters corresponded to *Pax3*::*Foxo1* in one of three lineages (*Myf5*, *Myf6*, and *Pax3*) while all tumours in MR1 corresponded to DNA alterations other than *Pax3*::*Foxo1* in these same three lineages. Finally, tumours in MR2 corresponded to either *Pax3*::*Foxo1* in the *Pax7* lineage or DNA alterations other than *Pax3*::*Foxo1* in one of three lineages (*Myf6*, *Pax3*, or *Pax7*). Dimensionality reduction analysis using t‐SNE yielded similar results (Figure 1B).

The unsupervised clustering distinguished the GEMMs in which *Pax3*::*Foxo1* was targeted to the *Myf5*, *My6*, or *Pax3* lineage from the GEMMs in which *Pax3*::*Foxo1* was targeted to the *Pax7* lineage. We previously demonstrated weak *Pax3*::*Foxo1* RNA expression in *Pax7* CreER tumours with the *Pax3*::*Foxo1* knock‐in allele compared to much higher *Pax3*::*Foxo1* expression in tumours forming after *Pax3*::*Foxo1* was knocked into the other lineages [17]. We concluded that the ML cluster consisted of mouse tumours with high *Pax3*::*Foxo1* expression (11/11, 100%), whereas the MR cluster consisted of mouse tumours with either no or weak *Pax3*::*Foxo1* expression (0/20, 0%) (*p* < 0.001) (Figure 1A). This observation suggests that the ML and MR clusters can be viewed as FP and FN mouse RMS tumours respectively. Furthermore, we observed that all the tumours with weak expression of *Pax3*::*Foxo1* were significantly enriched in the MR2 subset compared with the MR1 subset (*p* = 0.015). As the MR2 subset contains *Pax7* lineage tumours both with and without *Pax3*::*Foxo1* knock‐in, these findings further reinforce the notion that the functional and epigenetic impact of *Pax3*::*Foxo1* in the *Pax7* lineage is very limited [17]. As only one tumour arose in the setting of *Pax3*::*Foxo1* directed to the *Myf5* lineage, which is often embryonic lethal [17], the usual impact of *Pax3*::*Foxo1* on DNA methylation in this lineage is unclear.

We examined associations of the other driver mutations with the DNA methylation pattern (Figure 1A). We observed an enrichment of *Ptch1* mutations in the MR cluster (45%, 9/20) compared with the ML cluster (0%, 0/11) (*p* = 0.012). This association should be interpreted with caution as this finding may represent a selection bias since the *Ptch1* mutation was not incorporated into the GEMMs carrying the *Pax3*::*Foxo1* fusion gene. Furthermore, we did not observe any enrichment of the *Ptch1* mutation in either MR1 or MR2 subsets of the MR cluster when these two subsets were compared (*p* = 0.064). We did not observe statistically significant enrichment of the *RB1* mutation in either the ML or MR cluster (*p* = 0.317) or the MR1 or MR2 subset of the MR cluster (*p* = 0.494). The relationship of *Trp53* mutation with DNA methylation patterns could not be analysed because *Trp53* inactivation was present in all of the GEMMs.

We next studied the association between the two main DNA methylation clusters (ML and MR) and developmental lineage. Our analysis demonstrated that the *Pax7* lineage was tightly associated with mouse tumours in the MR cluster (9/20, 45%) compared with that in the ML cluster (0/11, 0%) (*p* = 0.012). This finding appears to be related to the weak expression of *Pax3*::*Foxo1* when activated in the *Pax7* lineage. In contrast, no statistically significant enrichments of the *Pax3* (*p* = 0.638) and *Myf5* (*p* = 0.631) lineages were observed between the ML and MR clusters (Figure 1A).

We found that *Myf6* lineage tumours were more prevalent in the ML cluster (7/11, 63.6%) than in the MR cluster (4/20, 20%) (*p* = 0.023). However, this association must be viewed with caution as this experimental cohort contained more *Myf6* lineage tumours with *Pax3*::*Foxo1* knock‐in than *Myf6* lineage tumours without *Pax3*::*Foxo1* knock‐in, and thus the prevalence of *Myf6* lineage tumours in the ML cluster may be attributable to selection bias. To further evaluate the enrichment of the *Myf6* lineage in the MR cluster, we used Cohen's kappa (*κ*) to evaluate the concordance between the presence of the *Myf6* lineage and high expression of *Pax3*::*Foxo1*. Overall, moderate agreement (*κ* = 0.44, 95% CI: 0.11–0.77) was demonstrated between the presence of *Myf6* lineage and high *Pax3*::*Foxo1* expression, implicating an interaction between *Myf6* lineage and *Pax3*::*Foxo1* during the development of these FP RMS tumours.

To further analyse the influence of lineage on the DNA methylation patterns in these mouse RMS tumours, we investigated the distribution of the four lineages between the two subsets (MR1 and MR2) within the MR cluster. GEMMs with the *Pax7* lineage showed dominant enrichment in the MR2 subset (9/12, 75%) and complete absence in the MR1 subset (0/8, 0%) (*p* = 0.001). In contrast, tumours with the *Myf5* lineage were exclusively enriched in the MR1 subset (4/8, 50%) and were absent in the MR2 subset (0/12, 0%) (*p* = 0.014). Statistically significant enrichments of the *Pax3* and *Myf6* lineages were not identified between the MR1 and MR2 subsets (Figure 1A). Collectively, these data demonstrated lineage differences between the two methylation‐defined subsets in the MR cluster: MR1 exhibiting *Myf5* lineage enrichment and MR2 exhibiting *Pax7* lineage enrichment.

### Comparison of DNA methylation in mouse and human RMS tumours

We investigated whether the DNA methylation patterns revealed in the GEMMs of RMS were similar to DNA methylation patterns in human RMS. To explore this possibility, we used previously generated DNA methylation data from *n* = 86 human RMS tumours [9]. We specifically selected probe sets from the mouse and human methylation platforms that target syntenic regions between human and mouse and, thus, allow direct comparison of DNA methylation patterns in human and mouse tumours [30]. Unsupervised hierarchical clustering analysis of mouse tumours with the syntenic probes identified clusters that were highly similar to the original clusters found in the analysis with all mouse array probes (supplementary material, Figure S1A). This similarity was confirmed in the t‐SNE analysis (supplementary material, Figure S1B). These results indicate that syntenic probes can be used as a surrogate for all mouse array probes to study DNA methylation patterns.

**Figure 2:**
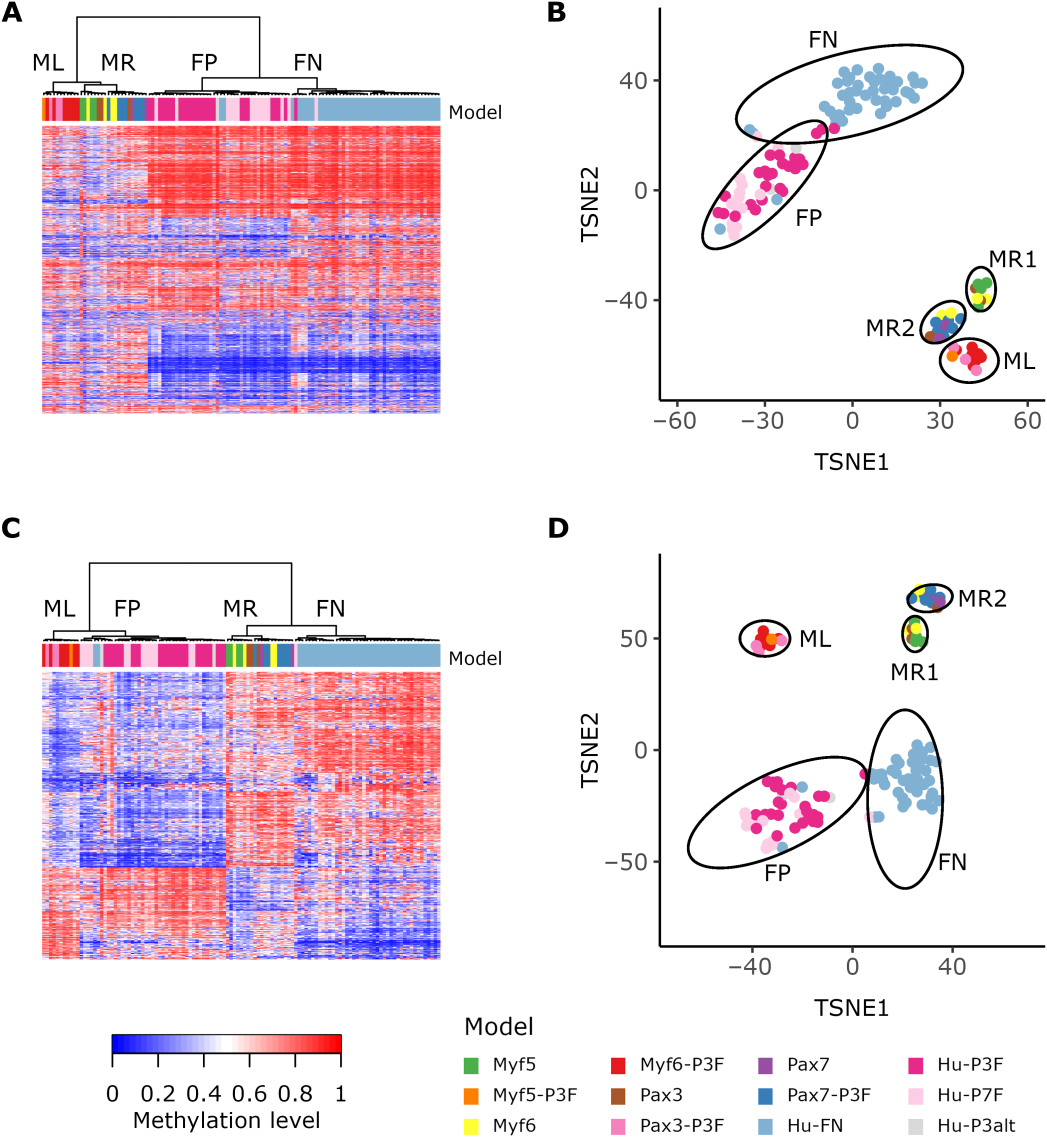
Association of DNA methylation patterns between RMS tumours in GEMMs and human RMS tumours. Unsupervised hierarchical clustering (A) and t‐SNE visualisation (B) of all mouse and human RMS tumours using the top 3,000 most variable syntenic CpG sites across all mouse tumours. Unsupervised hierarchical clustering (C) and t‐SNE visualisation (D) of all mouse and human RMS tumours, using the genes that are differentially methylated both between mouse ML and MR cluster tumours and between human FP and FN tumours. In panels (A) and (C), the dendrogram at the top shows the mouse tumours (ML and MR) and the human tumours (FP and FN). Beneath the dendrogram, the top‐row colours are based on the GEMM and/or human fusion subtype (defined in ‘Model’ key at bottom). In panels (B) and (D), each sample's colour is based on the GEMM and/or human fusion subtype (defined in ‘Model’ key at bottom).

We next asked whether the DNA methylation patterns identified in GEMM RMS tumours were related to the DNA methylation patterns found in human RMS. In extended clustering and t‐SNE analyses using the syntenic probe set in a combined cohort of human and mouse RMS tumours, the human FP and FN RMS tumours each formed separate clusters, and the mouse FP and FN RMS tumours also formed distinct clusters (Figure 2A,B). However, this DNA methylation analysis clearly demonstrated a dominant effect in which the mouse tumours, as a group, cluster separately from the human tumours, probably due to species‐specific DNA methylation effects.

We hypothesised the existence of intrinsic DNA methylation patterns that are common to FP and FN subsets in mouse and human RMS tumours. To test this possibility, we performed hierarchical clustering of human and mouse RMS tumours using genes that are differentially methylated (DM) between FP and FN subtypes in both human and mouse tumours (described in the following section). Our hierarchical clustering analysis showed two major groups of tumours: one group containing a combination of mouse and human FP tumours and a second group containing a combination of mouse and human FN tumours (Figure 2C). A t‐SNE visualisation study revealed a clear separation of FP and FN tumours along one axis as well as separation of mouse and human tumours along the second axis (Figure 2D). Taken together, these findings revealed similarities in DNA methylation patterns across species, supporting the hypothesis of conserved intrinsic DNA methylation features between FP and FN RMS tumours.

### DNA methylation differences between FP and FN subtypes in human and mouse tumours

**Figure 3:**
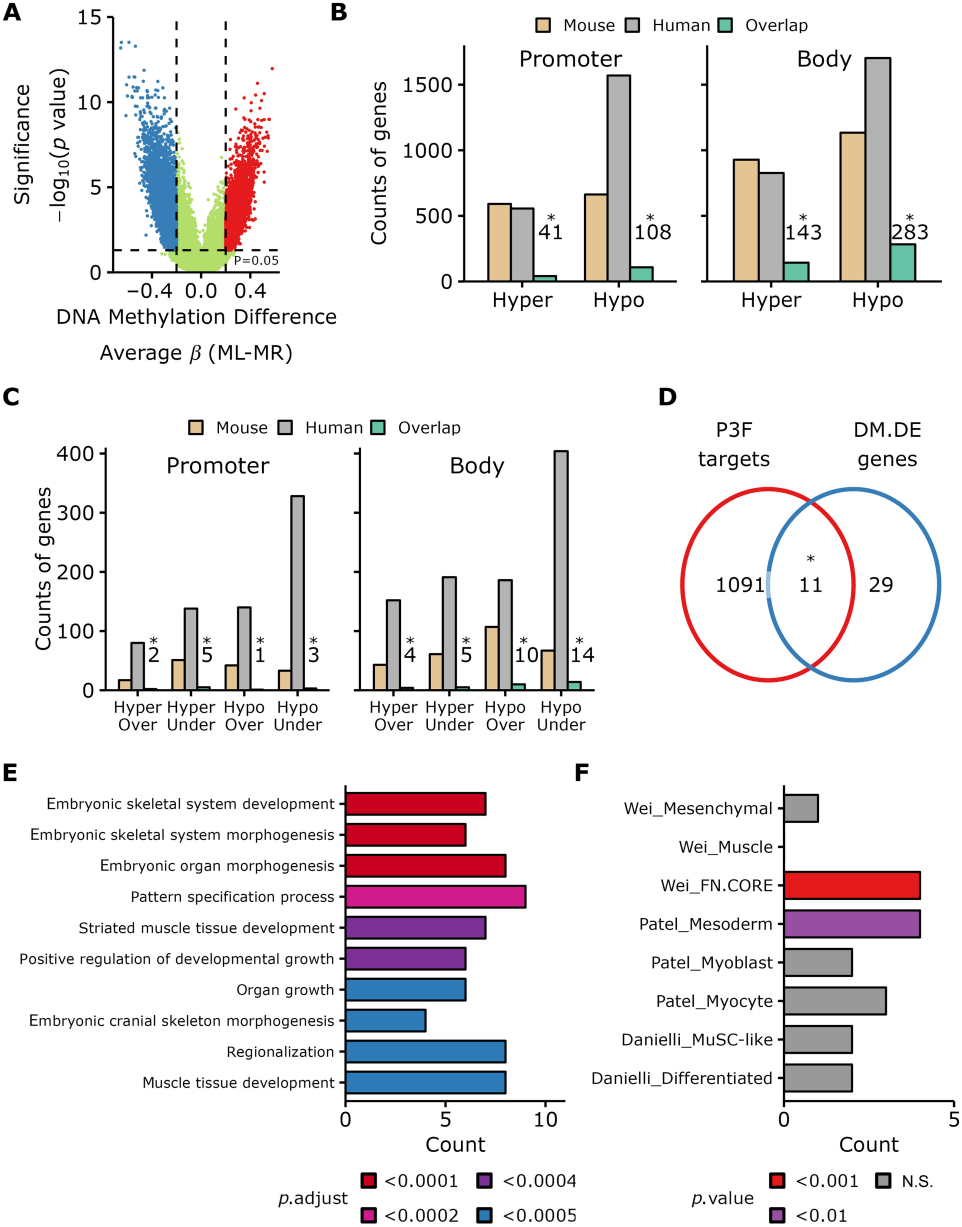
Characterisation of DM and DE genes between mouse ML and MR tumours and between human FP and FN RMS tumours. (A) Volcano plot depicting DM CpG sites. Probes that are significantly different between ML cluster and MR cluster (|beta| >0.2, adjusted *p* value <0.05) are in red (hypermethylated in ML cluster) or blue (hypomethylated in ML cluster). (B) Bar charts depicting DM genes (from reference gene set) in mouse (ML versus MR) and human (FP versus FN) RMS tumours and overlap between these two gene sets. (C) Bar chart depicting DM/DE genes (from reference gene set) in mouse and human RMS tumours and overlap between these two gene sets. (D) Venn diagram comparing DM/DE genes in both mouse and human RMS tumours with PAX3::FOXO1‐regulated targets defined by Cao *et al* [32] and Gryder *et al* [33] (*p* < 0.05). The asterisk denotes a significant overlap (*p* < 0.05, Fisher's test). (E) Top 10 Gene Ontology terms significantly enriched among DM/DE genes in mouse and human RMS tumours. (F) Overlap of expression profiles of RMS subpopulations defined by single‐cell sequencing studies [34, 35, 36] among DM/DE genes in mouse and human RMS tumours. In panels (E) and (F), the *x*‐axis indicates the number of enriched genes. Hyper, hypermethylated; Hypo, hypomethylated; Over, overexpressed; Under, underexpressed.

Given that ML and MR clusters correspond to mouse FP and FN tumours, respectively, we investigated whether differences in DNA methylation between ML and MR clusters in mouse overlap with DNA methylation differences between human FP and FN RMS tumours. We separately determined the DNA methylation differences between mouse ML and MR clusters and between human FP and FN RMS tumours and noted whether these differences corresponded to hypomethylation or hypermethylation in the promoter or gene body (Figure 3A,B, supplementary material, Tables S1 and S2). To examine overlap between DM genes in both mouse and human tumours, only genes common to both human and mouse methylation array platforms were used as a reference gene set. Although this analysis may include Cytosine‐phosphate‐Guanine (CpG) sites from syntenic regions discussed earlier, the focus in this analysis was on gene promoters or gene bodies that were represented on both arrays. The analysis revealed significant overlap in genes with hypomethylation or hypermethylation in the promoter or gene body between the mouse FP and FN tumours and between the human FP and FN tumours (range: 41–283, *p* < 0.01, Figure 3B).

### DNA methylation and expression differences between FP and FN tumours

We extended our analysis to the RNA expression level to assess the functional relevance of DNA methylation differences between the mouse ML and MR clusters. For this analysis, we used the gene expression dataset previously generated with the MouseRef‐8 Expression Beadchip [16, 17]. Based on their genotypes, we identified *n* = 23 tumours corresponding to the ML cluster (*Pax3*::*Foxo1* in *Myf5*, *Myf6*, or *Pax3* lineage) and *n* = 33 tumours corresponding to the MR cluster (other mutations in any lineage or *Pax3*::*Foxo1* in *Pax7* lineage) [16, 17]. Differential expression analysis identified 1,310 differentially expressed (DE) genes between mouse ML and MR tumours (supplementary material, Table S3). Comparison of DM genes and DE genes between ML and MR clusters identified a subset of genes that are both DM and DE; these genes were stratified according to hypomethylation or hypermethylation in the promoter or gene body (range: 17–107, Figure 3C).

We similarly determined DM/DE genes in human FP versus FN RMS tumours. Utilising a previously published RNA‐seq dataset from 38 FP and 67 FN human RMS tumours [4, 31], our differential expression analysis identified 5,116 DE genes in human FP versus FN tumours (supplementary material, Table S4). We then determined the genes that are both DM and DE between human FP and FN tumours and stratified these genes according to hypomethylation or hypermethylation in the promoter or gene body (range: 80–404, Figure 3C).

Finally, we compared the DM/DE genes between mouse ML and MR tumours and between human FP and FN tumours and found a total of 40 common DM/DE genes (matched by gene symbol) in both mouse and human RMS tumours (Table 2). To investigate an association between these DM/DE genes and *Pax3*::*Foxo1* regulation, we compared these 40 genes with the previously published lists of Pax3::Foxo1‐binding targets [32, 33]. Eleven out of the 40 genes were defined as Pax3::Foxo1 targets and were significantly enriched in this group of DM/DE genes (*p* < 0.02) (Table 2 and Figure 3D). Of note, some of these Pax3::Foxo1 target genes are hypomethylated and others are hypermethylated between FP and FN tumours, and similarly some are underexpressed and some are overexpressed between these tumour groups. These findings point to a conserved dynamic interaction between Pax3::Foxo1 action and DNA methylation, in which we hypothesise that DNA methylation may modulate the ability of Pax3::Foxo1 to express its target genes.

**Table 2:** Differentially methylated/differentially expressed (DM/DE) genes in mouse ML versus MR comparison and human fusion‐positive (FP) versus fusion‐negative (FN) comparison.

Gene symbol	Region	Methyl	Expres	P3F targets	Patel	Wei	Danielli
ABLIM2	body	hypo	over		Myoc		
ACAT1	body	hypo	over				
ACVR1C	body	hyper	over				
ANKRD50	body	hyper	under				
BCAT1	body	hypo	under	TRUE	Myoc		
BNC2	body	hyper	under		Meso		
CACNA1C	body	hypo	under		Meso		
CDH4	body	hyper	over	TRUE	Myob		
COL1A2	prom	hyper	under		Meso	Mese	MuSC, Diff
CRABP1	body	hypo	under				
DLL1	body	hypo	over				
DTNA	body	hypo	under		Myoc		
EMILIN1	prom	hyper	under			Fnco	Diff
EPHA8	body	hyper	over				
ERRFI1	body	hypo	over	TRUE			MuSC
ESPN	body	hyper	over				
FGF8	body	hypo	over	TRUE			
FMNL2	body	hypo	under				
GRIP1	prom	hyper	over	TRUE	Myob		
HOXC6	body	hyper	under			Fnco	
HOXD4	prom	hypo	under			Fnco	
	body	hypo	under			Fnco	
HOXD9	body	hypo	under				
IGF1	body	hypo	under				
IRX3	body	hypo	over				
KCNJ12	body	hypo	over				
KCNK2	prom	hyper	over				
	body	hypo	over				
LTBP4	prom	hypo	under			Fnco	
	body	hypo	under			Fnco	
MAN1C1	body	hypo	over	TRUE			
MXRA8	prom	hyper	under				
NEDD4L	prom	hypo	under	TRUE			
OLFML3	body	hyper	under				
OSBPL3	body	hypo	under				
RGMA	prom	hyper	under	TRUE			
	body	hyper	under	TRUE			
ROR2	body	hypo	under		Meso		
SCARA5	prom	hypo	over	TRUE			
SMAD3	body	hypo	under				
STK39	body	hypo	under				
TBX15	body	hypo	over	TRUE			
TGFBR2	body	hypo	under				
ZIC1	prom	hyper	under	TRUE			

*Note*: Patel, Wei and Danielli refer to gene sets derived from single cell studies in RMS [34, 35, 36].

Abbreviations: Cycl, cycling progenitors; Diff, differentiated; Expres, expression; Fnco, FN‐core; hyper, hypermethylation; hypo, hypomethylation; Mese, mesenchymal; Meso, mesoderm; Methyl, methylation; Mus, muscle; MuSC, muscle stem‐like; Myob, myoblast; Myoc, myocyte; over, overexpression; prom, promoter; under, underexpression.

Next, we used Gene Ontology (GO) analysis to identify gene categories that are biologically important among these 40 DM/DE genes. This analysis showed that the top 10 significantly enriched GO terms based on biological processes were related to development, highlighting conserved differences in normal developmental pathways between FP and FN RMS tumours in both the mouse and human (Figure 3E and supplementary material, Table S5). We further explored this phenomenon by comparing these 40 genes with several gene sets that are characteristic of muscle development and define RMS subpopulations in recently published single‐cell transcriptomic studies. Intriguingly, two of eight gene sets, Wei_FN.CORE and Patel_Mesoderm, were significantly enriched (Figure 3F and Table 2) [34, 35, 36]. For each of these two gene sets, the genes of interest were more highly expressed in FN mouse and human tumours, supporting the premise that features of these subpopulations related to muscle development overlap with the conserved features of FN tumours in both mouse and human.

### DNA methylation in mouse FN tumours with *Myf5* lineage versus *Pax7* lineage

**Figure 4:**
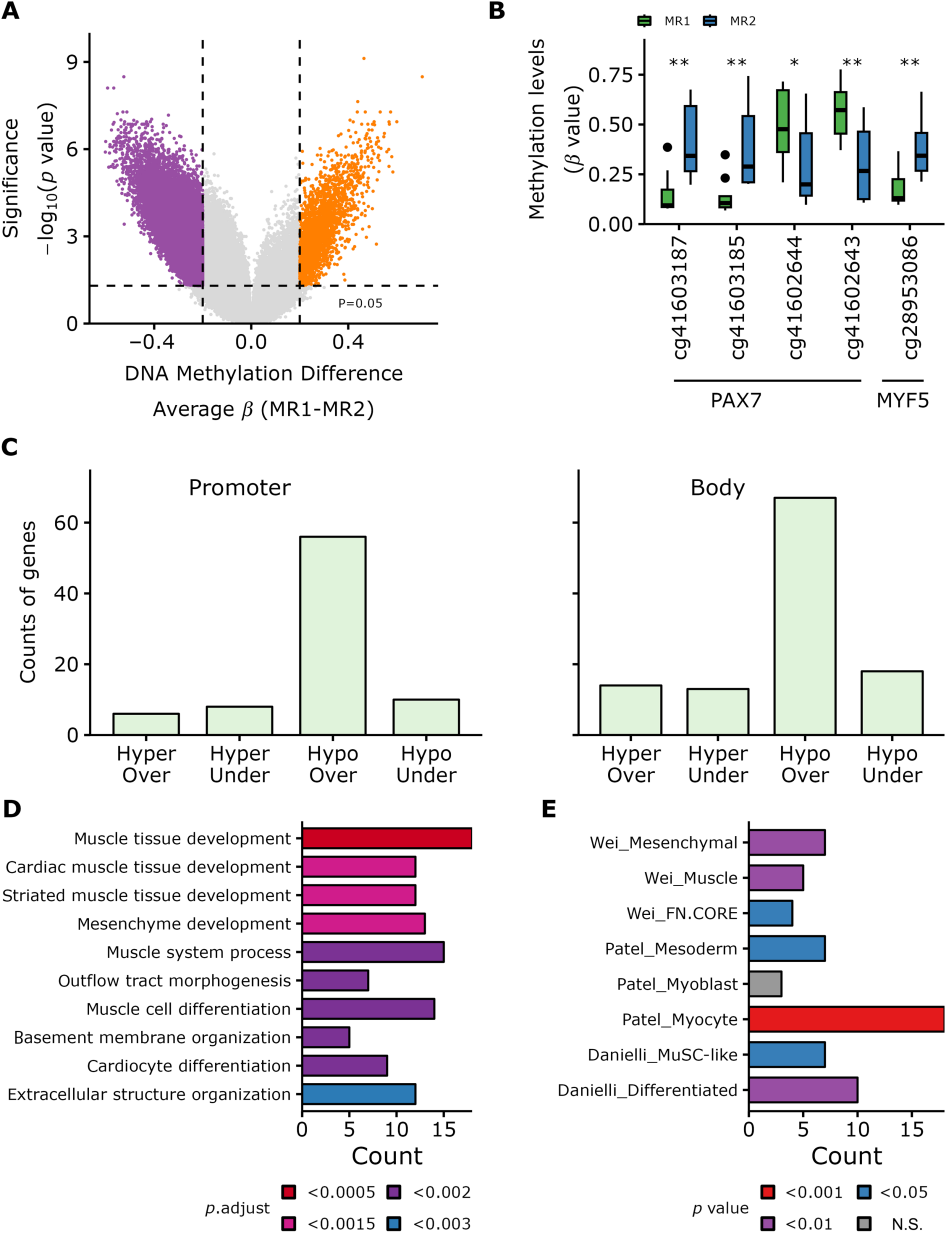
Characterisation of DM and DE genes between MR1 and MR2 subsets in genetically engineered mouse models of RMS. (A) Volcano plot depicting DM CpG sites. Probes that are significantly different between MR1 and MR2 (|beta| >0.2, adjusted *p* value <0.05) are coloured in orange (hypermethylated in MR1 cluster) or purple (hypomethylated in MR1 cluster). (B) Differential methylation of CpG sites in *Pax7* and *Myf5* genes in mouse RMS tumours from *Pax7* and *Myf5* lineage tumours. (C) Bar chart depicting DM/DE genes between MR1 and MR2 subsets. (D) Top 10 Gene Ontology terms significantly enriched among DM/DE genes between MR1 and MR2 subsets. (E) Overlap of expression profiles of RMS subpopulations defined by single‐cell sequencing studies [34, 35, 36] among DM/DE genes between MR1 and MR2 subsets. In panels (D) and (E), the *x*‐axis indicates the number of enriched genes. Hyper, hypermethylated; Hypo, hypomethylated; Over, overexpressed; Under, underexpressed.

We explored DNA methylation differences between the MR1 and MR2 subsets and identified differential hypomethylation and hypermethylation in the promoter and gene body (Figure 4A and supplementary material, Table S6). Based on the prevalence of *Myf5* and *Pax7* lineages in MR1 and MR2, respectively, we examined whether the *Myf5* and *Pax7* genes were DM between MR1 and MR2 tumours. *Pax7* displayed significant hypomethylation in the promoter region and significant hypermethylation in the body region in MR1 compared with MR2 (Figure 4B). Meanwhile, *Myf5* displayed significant hypomethylation in the promoter region in MR1 compared with MR2 (Figure 4B). These findings indicate that DNA methylation of these myogenic markers is associated with the lineage from which these tumours originated. In contrast, our previous analysis of *Myf5* and *Pax7* expression in these tumours indicated that the expression status of these myogenic markers did not reflect the cell of origin [16]; this finding suggests that expression phenotype is not fully synonymous with cellular origin.

We extended our analysis of differences between MR1 and MR2 subsets to the RNA expression level. In particular, we compared differential expression between four *Myf5* lineage tumours and 10 *Pax7* lineage tumours using available gene expression data (supplementary material, Table S7). We further compared the DM genes (involving the promoter or gene body) between MR1 and MR2 and the DE genes between the corresponding *Myf5* and *Pax7* subsets. Our analysis yielded a total of 153 DM/DE genes between *Myf5* lineage and *Pax7* lineage tumours (Figure 4C, Table 3 and supplementary material, Table S8). Of note, most of these DM/DE genes were hypomethylated and overexpressed in the MR1/*Myf5* compared to the MR2/*Pax7* tumours. GO enrichment analysis revealed that these DM/DE genes were significantly enriched in muscle‐related biological processes (Figure 4D and supplementary material, Table S9). These findings thus suggest that DNA methylation and associated changes in gene expression contribute to the inherent differences in muscle development‐related features between *Myf5* and *Pax7* lineage tumours. To further confirm this possibility, we compared our DM/DE genes with gene sets defined by single‐cell transcriptomic studies as described earlier [34, 35, 36]. Notably, seven out of eight muscle development‐related categories displayed significant statistical enrichment (Figure 4E and Table 3); this issue is particularly well illustrated by the finding of 16 genes associated with the myocyte signature that are more highly expressed in *Myf5* lineage tumours compared to *Pax7* lineage tumours. These overall findings thus support the importance of DM/DE muscle development genes in the biological differences between these two categories of mouse FN RMS tumours.

**Table 3:** Representative differentially methylated/differentially expressed (DM/DE) genes in mouse MR1 versus MR2 comparison.

Gene symbol	Region	Methyl	Expres	Patel	Wei	Danielli
ABLIM2	body	hypo	over	Myoc		
ABLIM3	body	hypo	over	Myoc		
ACTC1	prom	hypo	over	Myoc	Mus	MuSC, Cycl, Diff
CACNA1S	prom	hypo	over	Myoc		
CACNA2D3	body	hypo	over	Myob		
CAV3	prom	hypo	over	Myoc		
CCND3	prom	hypo	over			MuSC
	body	hypo	over			MuSC
COL15A1	prom	hyper	over	Meso	Mese	
	body	hypo	over	Meso	Mese	
COL25A1	body	hyper	over	Myoc	Mus	
	body	hypo	over	Myoc	Mus	
COL4A1	body	hypo	over	Meso	Mese	Diff
CORO1C	prom	hyper	under	Myoc		
	body	hyper	under	Myoc		
DNMT1	body	hypo	under			MuSC
ELN	prom	hyper	over	Meso	Mese	
	body	hypo	over	Meso	Mese	
ENPP6	prom	hypo	over	Myoc		
EPHB3	prom	hypo	over		Fnco	
	body	hypo	over		Fnco	
EPS8	prom	hypo	under	Meso	Mese	
	body	hypo	under	Meso	Mese	
FZD4	body	hypo	over		Fnco	
FZD7	prom	hypo	over		Fnco	
GNAS	prom	hypo	over	Myob		
HIP1	body	hyper	over	Myoc		
HRC	prom	hypo	over			Diff
ISLR	body	hypo	over			MuSC, Diff
LAMC1	body	hypo	over	Meso	Mese	MuSC
LY6E	body	hyper	under		Fnco	
MAST4	body	hypo	under	Meso		
MYL1	prom	hypo	over	Myoc	Mus	MuSC, Cycl, Diff
	body	hypo	over	Myoc	Mus	MuSC, Cycl, Diff
NCALD	prom	hyper	over	Myoc		Diff
	prom	hypo	over	Myoc		Diff
	body	hypo	over	Myoc		Diff
NEB	prom	hypo	over	Myoc	Mus	MuSC, Diff
	body	hypo	over	Myoc	Mus	MuSC, Diff
NFIA	body	hypo	over	Myob		
PLXNA2	body	hyper	over	Myoc		
	body	hypo	over	Myoc		
PPP1R14A	body	hypo	over		Mese	
PRICKLE1	prom	hypo	over	Meso		
	body	hypo	over	Meso		
RYR1	body	hyper	over	Myoc	Mus	Diff
S100A4	prom	hyper	under		Mese	Diff
	body	hyper	under		Mese	Diff
SMOC1	body	hypo	over	Myoc		
SVIL	prom	hypo	over	Myoc		
	body	hyper	over	Myoc		
	body	hypo	over	Myoc		
TNFRSF19	body	hypo	over	Myoc		
UBE2E3	prom	hypo	under	Myoc		Diff

*Note*: Patel, Wei, and Dannielli refer to gene sets derived from single‐cell studies in RMS [34, 35, 36].

Abbreviations: Cycl, cycling progenitors; Diff, differentiated; Expres, expression; Fnco, FN‐core; hyper, hypermethylation; hypo, hypomethylation; Mese, mesenchymal; Meso, mesoderm; Methyl, methylation; Mus, muscle; MuSC, muscle stem‐like; Myob, myoblast; Myoc, myocyte; over, overexpression; prom, promoter; under, underexpression.

## Discussion

Using RMS tumours arising in these GEMMs, our study demonstrates a strong association between Pax3::Foxo1 expression and DNA methylation pattern. This finding supports the hypothesis that the Pax3::Foxo1 fusion protein modulates the epigenetic landscape within several myogenic lineages. Although there is cell cycle regulation of Pax3::Foxo1 expression resulting in intercellular heterogeneity within these mouse FP RMS tumours, our results suggest that the overall long‐term Pax3::Foxo1 expression pattern contributes to DNA methylation [37]. In contrast, RMS tumours in which a *Pax3*::*Foxo1* fusion is directed to the *Pax7* lineage do not demonstrate differing methylation from RMS tumours in which other mutations are directed to the *Pax7* lineage. This latter finding can be explained by the very low expression of *Pax3::Foxo1* in these *Pax7* lineage tumours, resulting in an inadequate amount of Pax3::Foxo1 to modulate the epigenetic landscape in this setting. To confirm the effect of the Pax3::Foxo1 fusion protein on DNA methylation, knockout of *PAX3*::*FOXO1* in RH30 FP RMS cells resulted in marked differences in DNA methylation patterns compared to control RH30 cells (unpublished results). When expressed at high levels, Pax3::Foxo1 may modulate DNA methylation and other aspects of the epigenetic landscape by activating expression of downstream target genes such as *JARID2*, whose product interacts with the PRC2, which in turn interacts with DNA methyltransferases (DNMTs) [38, 39]. In a second possible scenario, Pax3::Foxo1 may influence DNA methylation through its interactions with epigenetic coregulators such as CHD4, a peripheral component of the NuRD complex that cooperates with DNMTs to maintain silencing of tumour suppressor genes [40, 41, 42]. Of note, expression of DNMTs was found to be lower in alveolar RMS tumours (predominantly FP) compared with embryonal RMS tumours (generally FN) [43, 44], and thus we propose that PAX3::FOXO1 may contribute to this expression difference and thereby influence the DNA methylation pattern. In addition to these possibilities, we also acknowledge the alternative hypothesis that DNA methylation alterations may be selected during multistep RMS development to collaborate with the *Pax3*::*Foxo1* fusion.

In mouse FN RMS tumours (with low or no *Pax3*::*Foxo1*), our study did not identify an association between other driver mutations and DNA methylation. However, our study does provide evidence for an association between the myogenic lineage and DNA methylation pattern. Starting with the finding that the DNMT inhibitor 5‐azacytidine induces myogenic differentiation of murine C3H10T1/2 fibroblasts [45], multiple studies have found an association between the myogenesis process and DNA methylation. During myogenic development, the muscle‐specific transcription factors, which comprise *Myf5*, *Myod1*, *Myog*, and *Myf6*, undergo a regulated programme of spatial and temporal expression [46]. Support for a role of DNA methylation in regulating myogenesis is provided by the finding that a distal enhancer in the *Myod1* gene is specifically demethylated *in vivo* during mouse somitogenesis in cells of the myogenic lineage [47]. In addition, the promoter of *Myog* is initially methylated in developing somites prior to *Myog* activation and is subsequently demethylated upon onset of myogenesis [48]. Finally, more recent studies revealed that *de novo* DNA methylation occurs in the myoblast genome during differentiation into myotubes [49, 50].

Our finding of a striking difference in DNA methylation patterns between mouse RMS tumours in which driver mutations were directed to the *Myf5* or *Pax7* lineage is consistent with the dynamic events that occur during myogenic development. *Pax7* marks myogenic progenitor cells and regulates their behaviour and entry into the skeletal muscle differentiation programme [51]. During embryogenesis, *Pax7* is expressed in the central domain of the dermomyotome, whereas *Myf5* is the first myogenic transcription factor to be activated when myogenesis is initiated in the somite [52]. In postnatal muscle, *Pax7* is necessary for satellite cell specification, whereas *Myf5* is required for the regenerative capabilities of these myogenic satellite cells [53, 54]. In concert with these findings, the DM/DE genes between *Myf5* and *Pax7* lineage‐related tumours are associated with muscle development‐related gene subsets. We propose that the differing DNA methylation patterns in FN RMS tumours in which driver mutations are directed to different myogenic lineages represent DNA methylation signatures characteristic of these different lineages. Lineage‐specific DNA methylation changes may also occur and contribute to RMS tumour development, but the relevance to human disease needs to be further investigated.

Our study of the relationship of DNA methylation and gene expression confirmed our previous findings that only a subset of DNA methylation changes was associated with detectable gene expression changes. Other DNA methylation differences may be more reflective of the differences in the underlying epigenetic architecture. For those genes in which DNA methylation differences are associated with expression changes, we identified a small group of genes that are relevant to RMS development and may represent important similarities between mouse and human RMS. The identification of multiple Pax3::Foxo1 targets (e.g. *FGF8* [55]) among the common DM/DE genes suggests that the Pax3::Foxo1 protein, which is a functional transcription factor, may coordinate with promoter or gene body methylation, either directly or indirectly, to regulate expression of its downstream targets.

In summary, our study of DNA methylation in FP and FN RMS arising in GEMMs provided new evidence of the biological features contributing to the DNA methylation patterns in human RMS tumours. Both mutational changes (expression of the Pax3::Foxo1 fusion protein) and cell of origin (specific myogenic lineage in which the tumour developed) were identified as important factors contributing to the DNA methylation pattern of these tumours. We are currently working on single‐cell sequencing studies of mouse and human RMS tumours to compare these DNA methylation patterns with the cellular subsets within individual RMS tumours. These results will help decipher the role of DNA methylation in RMS biology and help focus attention on the epigenetic landscape to identify novel therapeutic targets for this childhood cancer.

## Author contributions statement

WS, CK and FGB conceived the project and designed the experiments. CK provided the mouse models. WS, SMH and HW performed the experiments. WS and FGB performed computational analyses. FGB supervised the research. WS and FGB wrote the manuscript with input from all authors.

## Supporting information


Supplementary materials and methods
**Figure S1.** DNA methylation analysis of genetically engineered mouse models (GEMMs) of rhabdomyosarcoma (RMS) using mouse/human syntenic gene set

**Table S1.** Differentially methylated probes in mouse ML versus MR tumours

**Table S2.** Differentially methylated probes in human FP versus FN tumours

**Table S3.** Differentially expressed genes in mouse ML versus MR tumours

**Table S4.** Differentially expressed genes in human FP versus FN tumours

**Table S5.** Top 10 significantly enriched terms based on biological processes among differentially methylated/expressed genes in mouse ML versus MR tumours and human FP versus FN tumours

**Table S6.** Differentially methylated probes in mouse myogenic factor 5 (Myf5) versus paired box 7 (Pax7) tumours

**Table S7.** Differentially expressed genes in mouse myogenic factor (Myf5) versus paired box 7 (Pax7) tumours

**Table S8.** Differentially methylated/expressed genes in mouse myogenic factor (Myf5) versus paired box 7 (Pax7) tumours

**Table S9.** Top 10 significantly enriched terms based on biological processes among differentially methylated/expressed genes in mouse myogenic factor (Myf5) versus paired box 7 (Pax7) tumours

## Data Availability

The data that support the findings of this study are available from the authors upon reasonable request. The raw DNA methylation data of GEMMs of RMS are publicly available in NCBI's Gene Expression Omnibus with accession number GSE260806 (https://www.ncbi.nlm.nih.gov/geo/query/acc.cgi?acc=GSE260806).
