# Supplementary material for: DNA methylation patterns are influenced by Pax3 :: Foxo1 expression and developmental lineage in rhabdomyosarcoma tumours forming in genetically engineered mouse models
Source: J Pathol. 2025 Jan 15;265(3):316–29. doi: 10.1002/path.6386 (PMC11794984; doi:10.1002/path.6386)
Supplement: Supplementary file 1 — Supplementary materials and methods Figure S1. DNA methylation analysis of genetically engineered mouse models (GEMMs) of rhabdomyosarcoma (RMS) using mouse/human syntenic gene set [file PATH-265-316-s003.docx]

**DNA methylation patterns are influenced by *Pax3*::*Foxo1* expression and developmental lineage in rhabdomyosarcoma tumours forming in genetically engineered mouse models**

W Sun *et al J Pathol* <https://doi.org/10.1002/path.6386>

**Supplementary materials** **and methods\**

**Supplementary Figure S1**

**Supplementary Tables S1–S9 are provided as separate Excel files**

Reference numbers refer to the main text list.

**Supplementary materials and methods**

**Genome-wide DNA methylation analysis**

Genomic DNA from mouse tumours was analysed on the Infinium Mouse Methylation BeadChip (Illumina, San Diego, CA, USA) (GEO: GSE260806). DNA methylation data from human RMS tumours previously generated on the Infinium HumanMethylation450 (HM450) BeadChip (Illumina) were included in the analysis (dbGaP: phs001970). DNA methylation data were normalised using the Subset-Quantile Within Array Normalization (SWAN) algorithm in the minfi package (http://bioconductor.org/packages/release/bioc/html/minfi.html, last accessed 22 April 2024) [26]. Probes with a detection *p* value >0.01 in at least one sample, probes located on the X or Y chromosome, and non-CpG probes were discarded. For HM450 analysis, probes containing a SNP at the single-base extension or CpG site, probes with genetic variants overlapping the body of the probes, and probes identified as cross-hybridising were also discarded [56,57]. The β value was computed as the measure of methylation, ranging from 0 (completely unmethylated) to 1.0 (completely methylated). Hierarchical clustering and t-Distributed Stochastic Neighbor Embedding (t-SNE) were generated using the gplots and t-SNE packages (https://CRAN.R-project.org/package=gplots and https://CRAN.R-project.org/package=Rtsne, last accessed 22 April 2024) [9,10,27].

Significant methylation of a probe was determined using the limma package (https://bioconductor.org/packages/limma, last accessed 22 April 2024) [58] with a threshold of |Δβ| ≥ 0.2 and Benjamini and Hochberg (BH)-adjusted *p* value <0.05. The statistical significance between molecular groups and DNA methylation clustering was determined using Fisher’s test with *p* < 0.05 considered significant.

**Gene expression analysis.** For mouse tumours, gene expression analysis was previously performed using MouseRef-8 Expression BeadChip (Illumina) [16,17]. For human tumours, RNAseq data were obtained from the OncoGenomics database (<https://pob.abcc.ncifrf.gov/cgibin/JK>, last accessed 22 April 2024) [28]. Differential expression analysis was performed using the limma package for mouse tumours and Student’s *t*-test for human tumours. Genes were considered differentially expressed based on a log2 fold-change >1.4 and a BH-adjusted *p* value <0.05. GO term overrepresentation analysis was performed using the clusterProfiler package (https://bioconductor.org/packages/release/bioc/html/clusterProfiler.html, last accessed 22 April 2024).

**
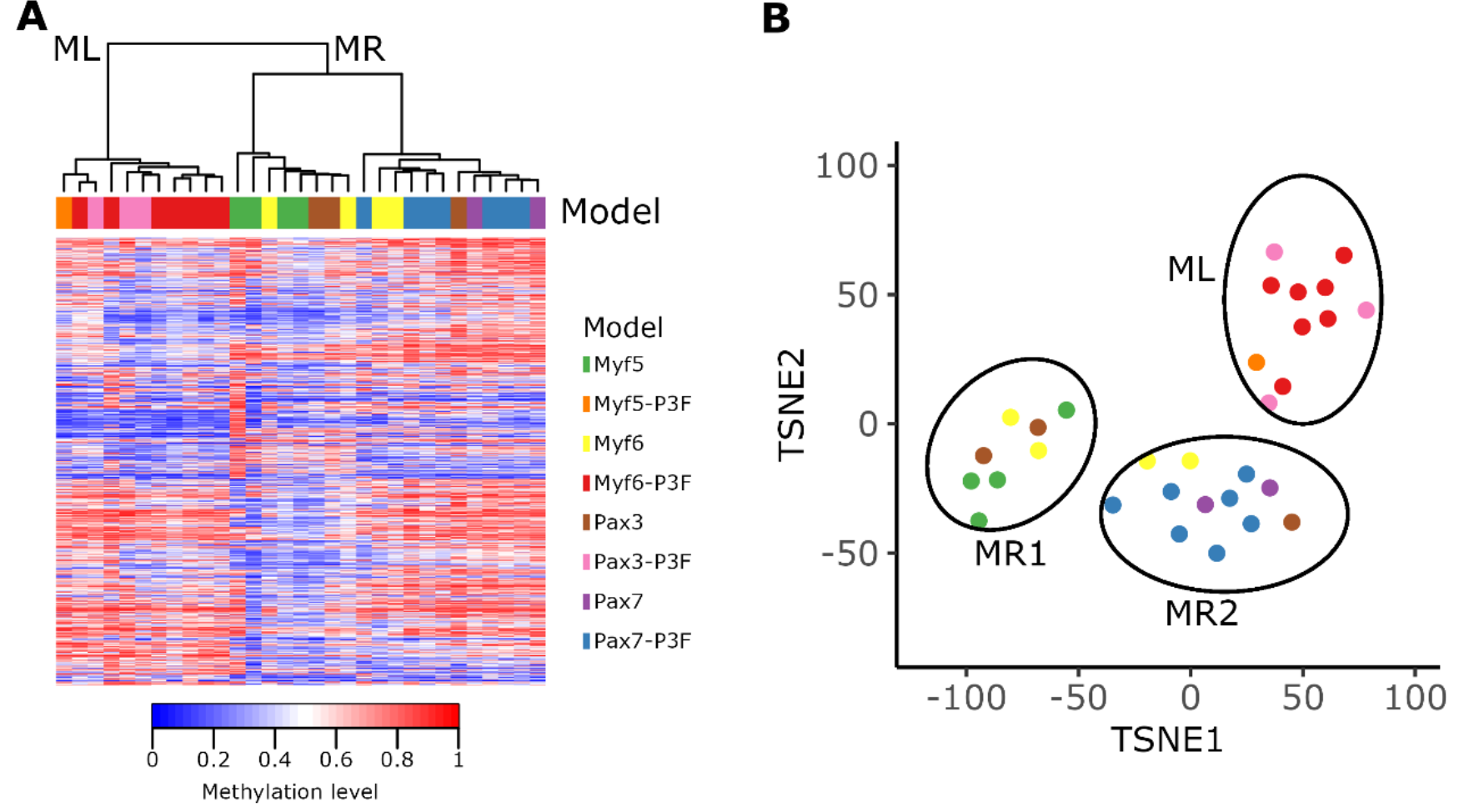
**

**Figure S1. DNA methylation analysis of genetically engineered mouse models (GEMMs) of rhabdomyosarcoma using mouse/human syntenic gene set.** (A) Unsupervised hierarchical clustering of methylation profiles from n=31 mouse tumours. The analysis used the top 3000 most variable probes across all mouse tumours selected from a probe set that targets syntenic regions between human and mouse samples. In the heatmap, rows represent probes and columns represent samples. The dendrogram at the top of panel (A) shows the main clusters (Methylation Left-cluster and Methylation Right-cluster, labelled as ML and MR, respectively). Immediately beneath the dendrogram, the row is coloured based on the genetically modified mouse models (GEMMs) defined in the “Model” key. (B) *t*-distributed Stochastic Neighbor Embedding (t-SNE) visualisation of all mouse tumours using probes described in part (A). Each sample is coloured based on the GEMMs defined in the legend in panel (A) and the groupings circled to indicate the ML, MR1 and MR2 categories in panel (B).
